# *Diplodia fraxini*: The Main Pathogen Involved in the Ash Dieback of *Fraxinus angustifolia* in Croatia

**DOI:** 10.3390/microorganisms13061238

**Published:** 2025-05-28

**Authors:** Jelena Kranjec Orlović, Carlo Bregant, Benedetto T. Linaldeddu, Lucio Montecchio, Ida Volenec, Katarina Uidl, Danko Diminić

**Affiliations:** 1 Institute of Forest Protection and Wildlife Management, University of Zagreb Faculty of Forestry and Wood Technology, Svetošimunska cesta 23, 10000 Zagreb, Croatia; kuidl@sumfak.unizg.hr (K.U.); ddiminic@sumfak.unizg.hr (D.D.); 2 Dipartimento Territorio e Sistemi Agro-Forestali, Università degli Studi di Padova, Viale dell’Università 16, 35020 Legnaro, Italy; benedetto.linaldeddu@unipd.it (B.T.L.); montecchio@unipd.it (L.M.); 3 Division for Forest Protection and Game Management, Croatian Forest Research Institute, Cvjetno naselje 41, 10450 Jastrebarsko, Croatia; idav@sumins.hr

**Keywords:** narrow-leaved ash, *Botryosphaeriaceae*, *Hymenoscyphus fraxineus*, *Diaporthe eres*, *Armillaria gallica*, *Lentinus tigrinus*

## Abstract

*Fraxinus angustifolia*, the main ash species in Croatia in terms of economic and ecological importance, is affected by a severe dieback initially attributed to the fungal pathogen *Hymenoscyphus fraxineus*. Recently, another pathogen, *Diplodia fraxini*, has been shown to play a key role in ash dieback in several European countries. Therefore, because the dieback symptoms of ash trees observed in Croatia are typical of *Botryosphaeriaceae* attacks, the aim of this study was to define the etiology of *F. angustifolia* dieback. To this end, symptomatic shoots and branches and cross-sections of the main stem were sampled from 20 symptomatic trees at eight locations and analyzed for the presence of *D. fraxini* and other possible fungal pathogens. *Diplodia fraxini* was the fungus most frequently associated with branch cankers and dieback; it was isolated from 17 trees in all sites monitored, and its pathogenicity towards *F. angustifolia* was confirmed. The fungus was also associated with wood necrosis at the base of the trunk in two trees. Other fungi, namely *H. fraxineus*, *Diaporthe eres*, *Diplodia seriata*, *Botryosphaeria dothidea*, *Armillaria gallica*, and *Lentinus tigrinus*, were isolated sporadically.

## 1. Introduction

*Fraxinus excelsior* L. and *F. angustifolia* Vahl, known as common ash and narrow-leaved ash, are widespread forest tree species with high economic and ecological significance in Europe, where they mostly occupy floodplain and riparian forest areas, but also urban areas and landscapes [1,2]. During the 1990s, an extensive *F. excelsior* dieback occurred across Poland, from where it spread to other European countries and where it is still affecting both ash species [3,4,5,6]. Symptoms on young and mature trees include leaf necroses, wilting and premature shedding of foliage, brown-to-orange bark necrosis and sunken cankers on twigs and branches, wood discolorations, and necroses in stems and roots [7,8]. The main role in causing these symptoms and the phenomenon of ash dieback in Europe has initially been attributed to the ascomycetous fungus *Hymenoscyphus fraxineus*, which was most probably introduced to Europe from Eastern Asia, where it is considered to be a native endophyte and leaf litter saprotroph on *Fraxinus* spp. [9,10]. According to data collected from different European countries, it is estimated that the ash dieback has caused ash mortality rates of up to 85% in plantations and 69% in natural stands [11].

Since the beginning of the phenomenon, the role of other potentially pathogenic fungi in the ash dieback has also been studied. Species of the genus *Armillaria*, as well as some other decay fungi (*Ganoderma* spp., *Bjerkandera adusta*, *Coprinellus* spp., etc.), were found to be mostly the secondary and in some cases even primary invaders of the weakened trees, which accelerate the dieback process by destroying the root system and stem base of infected trees [4,12,13,14,15,16,17]. Many potentially pathogenic fungal species were found in the stems and crowns of declining ash trees as well (*Diaporthe eres*, *Diplodia* spp., *Fusarium* spp., etc.), and their pathogenicity towards ash has been confirmed to a varying degree in artificial trials [8,18,19,20]. Recent studies demonstrated that the species *Diplodia fraxini* was the main species isolated from necrotic shoots and branches of both *F. angustifolia* and *F. excelsior.* The name *D. fraxini* was re-instated in 2014, when the study by Alves et al. [21] revealed that some of the *D. mutila*-like isolates from *Fraxinus* spp. should be assigned to this species, first described as *Sphaeria fraxini* by Saccardo in 1884 [22]. Reports from Portugal, Spain, Italy, and Slovenia indicate that *D. fraxini* is currently the main causative agent of branch cankers and dieback, being more aggressive to ash in comparison with *H. fraxineus* in artificial trials [23,24,25,26]. This is corroborated by the fact that *D. fraxini* is able to produce host-specific phytotoxins towards ash [27]. The fungus was also frequently associated with sunken cankers and mortality of *F. ornus* in Italy [28] and stem collar necroses on *F. excelsior* in Germany [14,15,29,30].

In Croatia, *F. angustifolia* is the main ash species in terms of growth area, volume of wood stock, and economic and ecological significance. It is the second most abundant floodplain tree species after *Quercus robur* L., occupying 3.06% of total forest area (72,690 ha) and participating with 3.19% in total wood growth stock (17,619,000 m^3^) [31]. It mostly grows in single-species forest stands; and in areas exposed to floods during long periods, where it acts as an important pioneer tree species. According to the data from the International Co-operative Programme on Assessment and Monitoring of Air Pollution Effects on Forests, known as ICP-Forests, the number of significantly defoliated *F. angustifolia* trees doubled in 2014 in comparison with previous years [32]. Since then, it has remained one of the most damaged forest tree species in Croatia, with 49.09 to 78.26% of trees displaying crown defoliation greater than 25% from year to year [32,33]. This pronounced decline followed soon after the confirmation of the pathogenic fungus *H. fraxineus* in crowns of symptomatic trees in 2011 in several locations across Croatia [34]. In 2017, the pathogen was confirmed in the roots and stem bases of *F. angustifolia* trees as well; however, the presence was far less frequently than other fungal species, such as *Ilyonectria robusta*, *Fusarium solani* and *Diaporthe cotoneastri*. Decay fungi (*Armillaria* spp., *Ganoderma adspersum*) were also found in some of the declining trees. It was concluded that the *F. angustifolia* decline in Croatia is driven by the interaction of several fungal species, rather than by *H. fraxineus* alone [4].

Given the new findings reporting the significance of *D. fraxini* in the ash decline in several countries, including the neighboring ones, the main goal of this study was to investigate the possible involvement of this aggressive pathogen in the *F. angustifolia* dieback in Croatia. The following aims were set accordingly: (1) to determine whether *D. fraxini* is present in the crowns and stem bases of declining trees of various ages and crown defoliation classes; (2) to determine to what amount *D. fraxini* is present in sampled plant tissues compared with other known ash fungal pathogens; and (3) to test the pathogenicity of the fungus towards *F. angustifolia*.

## 2. Materials and Methods

### 2.1. Field Sampling

**Figure 1:**
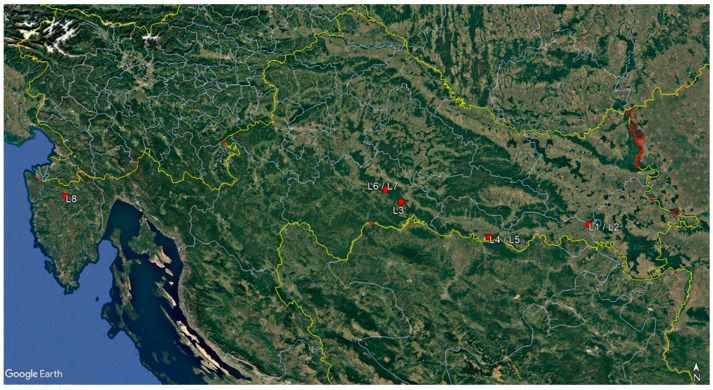
Map of sampling sites.

Monitoring surveys were conducted from October 2021 to December 2024 in eight *F. angustifolia* forest stands exhibiting typical symptoms of ash dieback. Except for one mixed forest stand of *Quercus robur* and *F. angustifolia* (site L4)*,* all the others were single-species forest stands with a more than a 75% share of *F. angustifolia* in the total wood stock (Table 1). Seven forest stands were in the Sava River Basin, where narrow-leaved ash occupies the largest continuous forest area (28,000 ha) in Croatia [35]. One forest stand was in the Istria Peninsula in the Mirna River valley (Figure 1). Two to four *F. angustifolia* trees were randomly selected and sampled in each forest stand (20 trees in total). Crown defoliation was assessed for each sampled tree, in accordance with the methodology of ICP-Forests [36].

For each tree, seven to ten shoots or branches exhibiting bark necrosis or sunken cankers were taken from the crown, as well as a cross-section of the stem base at a 30 cm height from the ground. In the laboratory, one to five subsamples of wood at the edges of present discolorations or necroses, approximately 7 × 7 cm in size, were taken from each stem base cross-section for further analysis (Table 1).

### 2.2. Fungal Isolation and Identification

Fungal isolations from symptomatic shoots and branches were attempted from the edges of inner bark (phloem) and wood (xylem) necroses. Sections of twigs and branches approximately 10 cm in length were surface-sterilized by soaking in 96% ethanol for 1 min, then in 4% sodium hypochlorite (NaOCl) for 5 min and again in 96% ethanol for 30 s. As for the sampled stem bases, fungal isolations were attempted only from the edges of wood (xylem) discolorations and necroses observed on the cross-sections taken. Pieces of wood approximately 7 × 7 cm^2^ in size, taken at the edges of discolorations or necroses, were surface-sterilized by immersing them in 96% ethanol for 10 s, followed by a rinse in sterile distilled water. Their number per cross-section varied depending on the appearance of symptoms (Table 1). Pieces of phloem and xylem (from shoots and branches), or only xylem (from stem base cross-sections), approximately 5 × 5 mm^2^ in size, were aseptically placed on malt extract agar (MEA, Oxoid, Hampshire, UK) supplemented with streptomycin sulphate (200 mg/L, Sigma-Aldrich, St. Louis, MI, USA). Plates were incubated in the dark at room temperature (20–21 °C) and they were monitored daily for three weeks. Emerging mycelia were regularly transferred to fresh MEA plates to obtain pure cultures. Cultures were initially grouped into morphotypes based on micromorphological features and colony growth patterns, observed after 7 days of incubation on potato dextrose agar (PDA, Oxoid) at 25 °C in the dark. At least one culture per morphotype and per sampled tree was subjected to molecular analysis to confirm its identity.

DNA was extracted from cultures grown in malt extract broth (MEB, Liofilchem, Roseto degli Abruzzi, Italy) in 2 mL microtubes for 7 days by the salting-out method according to Cenis [37], with minor modifications according to Kranjec Orlović et al. [38]. The internal transcribed spacer (ITS) region of the ribosomal DNA (rDNA) was amplified in a polymerase chain reaction (PCR) with primers ITS1-F [39] and ITS 4 [40] in 35 µL reactions containing 1X PCR buffer, 1.5 mM MgCl_2_, 0.2 mM dNTPs, 1 U of Taq polymerase (TaKaRa Taq™ DNA Polymerase, Takara Bio Inc., Kusatsu, Japan), 0.5 µM of each primer, 1 µL of DNA template (30–50 ng/µL), and sterile distilled water up to 35 µL. PCR conditions were as follows: an initial denaturation at 95 °C for 5 min, 35 cycles of denaturation at 95 °C for 30 s, annealing at 55 °C for 30 s, extension at 72 °C for 30 s, and a final extension step at 72 °C for 7 min. The resulting PCR products were sequenced using the same primers used for the amplification at the DNA sequencing facility of Macrogen Europe (Amsterdam, The Netherlands). After processing the raw data using BioEdit Sequence Alignment Editor v.7.2.5 software [41], sequences were identified by comparison with reference sequences of ex-type culture of each species available at the National Center for Biotechnology Information (NCBI) GenBank using the Basic Local Alignment Search Tool (BLAST + 2.15.0) tool [42]. New sequences were deposited and are available in GenBank (accession numbers PV490087-PV490132).

### 2.3. Pathogenicity Test

To confirm pathogenicity of *D. fraxini* towards *F. angustifolia*, an open-field trial was set up in the nursery “Šumski vrt i arboretum” located at the University of Zagreb Faculty of Forestry and Wood Technology (45.8202° N, 16.0228° E). Asymptomatic 6-year-old *F. angustifolia* trees grown in the ground from seeds were inoculated with two different *D. fraxini* isolates: (1) isolate D27 (GenBank: PV490126), obtained from wood necrosis at the stem base of 60-year-old *F. angustifolia* trees in location L8 (the Mirna River valley in Istria); and (2) isolate D44 (GenBank: PV490103), obtained from bark and wood necrosis in shoots of 26-year-old *F. angustifolia* trees in location L4 (the Sava River Basin). Twelve trees were inoculated with each isolate, and twelve control trees were mock-inoculated with sterile MEA plugs. Trees were on average 4.2 m (±0.1 m) tall and had an average breast height diameter (DBH) of 3.1 cm (±0.9 cm).

A wound was inflicted on the stems of trees at breast height (1.3 m), with a sterile steel cork borer (7 mm diameter). Bark was removed and a mycelium-MEA plug (6 mm diameter) taken from the margin of a 4-day-old pure culture was placed in the wound with mycelium facing the inner bark. The detached outer bark was replaced on top of the inserted plug, and the stem was wrapped with Parafilm (Bemis Company Inc., Neenah, WI, USA) and with a piece of aluminum foil. Control trees were inoculated with sterile MEA plugs following the same procedure.

During the trial, which was conducted from 10 June to 19 August 2024, the trees were exposed to natural outdoor conditions. A small data logger (DS1923-F5 Hygrochron Temperature and Humidity Data Logger, iButtonLink Technology, Whitewater, WI, USA) was placed on a wooden pole at a 2 m height to record air temperature and humidity during the trial. The occurrence and progression of symptoms on trees were checked two weeks after the inoculations and then monitored weekly until the end of the trial. Observed necroses were measured with an accuracy of 1 mm (both length and width).

The re-isolation of *D. fraxini* was attempted 10 weeks after inoculation. Stem sections approximately 15 cm long, containing an inoculation point, were submerged in 96% ethanol for 1 min, 4% sodium hypochlorite (NaOCl) for 5 min, and again in 96% ethanol for 30 s. Eight small pieces (5 × 5 mm^2^) of phloem and xylem per tree were aseptically taken from the margins of observed necrosis or from around the inoculation point in control trees, and they were placed on the MEA. Obtained cultures were identified as *D. fraxini* based on their morphological characteristics. The identification was confirmed by a molecular analysis of the ITS region of DNA, as already described, for one representative culture per tree.

## 3. Results

### 3.1. Observed Symptoms and Ocurrence of Diplodia Fraxini on Sampled Trees

**Figure 2:**
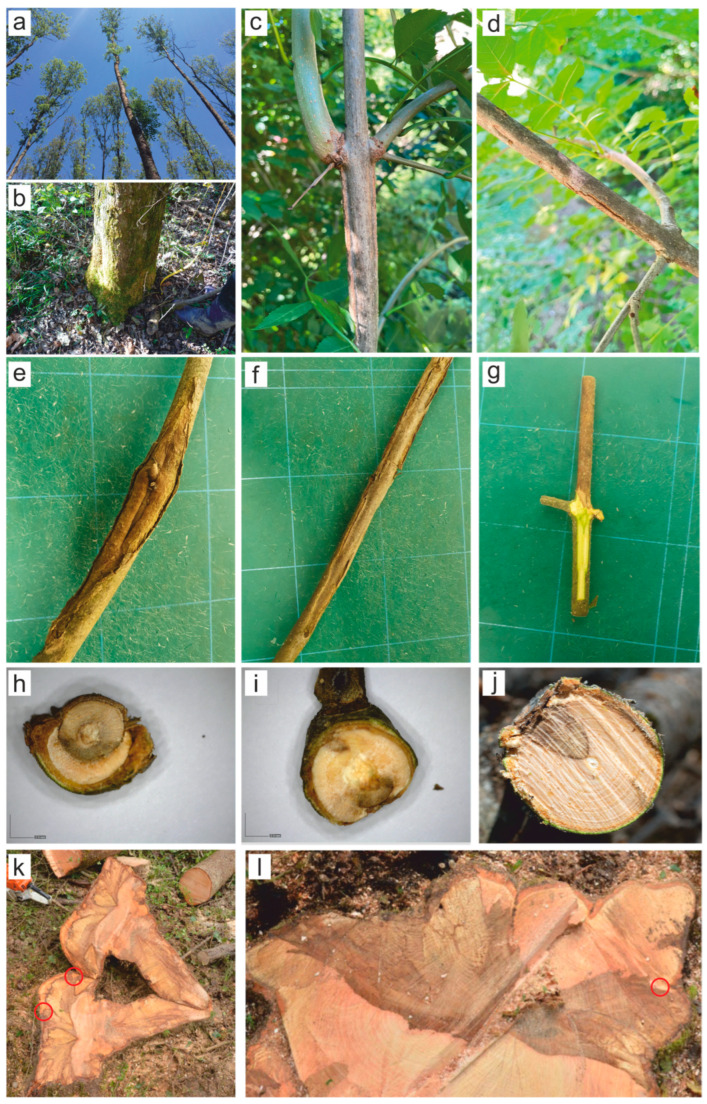
Major symptoms observed on sampled trees: (**a**) mature trees showing ash dieback in the studied forest stands; (**b**) lack of observable symptoms on the bark at the stem base of sampled trees; (**c**–**g**) bark necrosis and sunken cankers on shoots and branches; (**h**–**j**) V-shaped and circular necrotic sector visible on shoot and branch cross-sections; (**k**,**l**) stem base cross-sections of two trees in locations L8 (left) and L1 (right) from which *D. fraxini* was isolated; red circles are indicate an isolation point.

Typical symptoms of ash dieback (crown defoliation, shoot and branch cankers and dieback, epicormic shoots along the stem, increased tree mortality) were present in all the monitored sites (forest stands). Severe defoliation and dieback of shoots and branches were observed on all sampled trees to varying degrees. Brown to reddish bark discoloration as well as sunken cankers were visible on affected shoots and branches. These bark necroses extended from the outer bark to the phloem and xylem in a wedge- or circle-shaped formation. There were no observable outer symptoms on the bark (bark necrosis, cankers, lesions) at the stem base of sampled trees, except for tree No.2 in location L4, where an open wound (mechanical injury) was present. Nevertheless, wood discoloration and necrosis were observed on stem base cross-sections in 18 sampled trees to a various extent. In seven of these trees, rot was also present(Appendix A—Table A1, Figure 2).

*Diplodia fraxini* was the most frequently isolated species in this research; its occurrence was confirmed in all the locations studied and on 17 out of 20 sampled trees (Table 2). It occurred on trees 20 to 70 years old and with a crown defoliation varying between 20 and 70%. It was present in 65 of the 162 shoots and branches sampled. Furthermore, 5 out of 63 wood subsamples were positive for this pathogen (Appendix A—Table A1, Figure 2). The BLAST analysis of ITS sequences confirmed the identity of all 70 isolates of *D. fraxini*, with an identity value of 100% with the sequence of the ex-type culture of *D. fraxini* (CBS 136010) [21].

Six further species, namely *Diaporthe eres* (33 isolates), *Armillaria gallica* (9), *H. fraxineus* (10), *Lentinus tigrinus* (7), *Diplodia seriata* (2), and *Botryosphaeria dothidea* (1), were isolated from symptomatic samples showing inner bark necrotic lesions in one to fourteen trees (Table 2).

### 3.2. Pathogenicity of Diplodia fraxini on Fraxinus angustifolia

**Figure 3:**
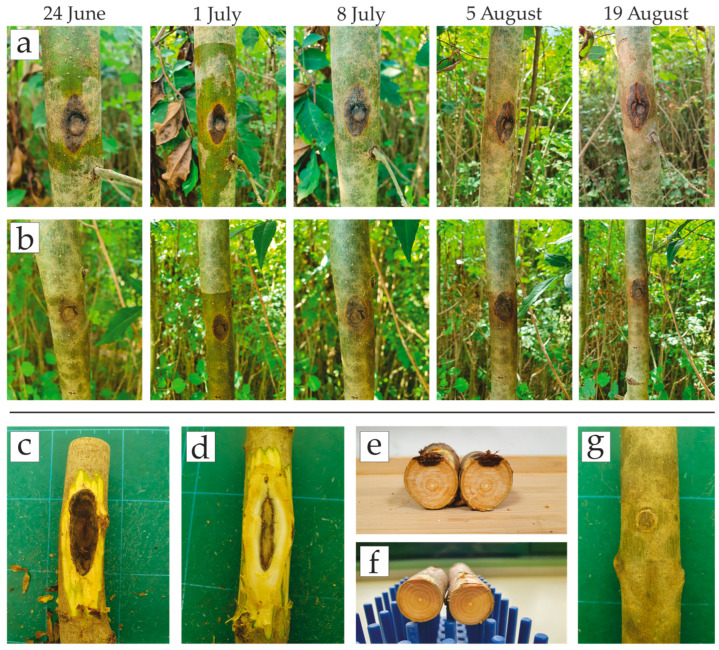
Results of the pathogenicity test on *F. angustifolia* trees inoculated with *D. fraxini* isolates: (**a**,**b**) progression of necroses caused by the isolate D27 (upper row) and D44 (lower row) on one of the inoculated trees during the trial; (**c**–**e**) extension of necroses underneath the outer bark into the phloem and xylem with a typical V-shaped section; (**f**,**g**) lack of symptoms on control trees.

In the pathogenicity test *D. fraxini* proved to be an aggressive pathogen on *F. angustifolia*, causing bark necrosis extending up and down from the inoculation points in all inoculated trees already visible during the first monitoring (two weeks after inoculation). Average length and width at this time was 15.6 mm and 10.7 mm for the isolate D27; and 15.4 mm and 11.3 mm for the isolate D44 (Table 3). By the end of the pathogenicity trial, bark necroses became dark brown in color with a typical sunken canker. The average length and width of the final necroses were 22.4 mm and 16.4 mm for the isolate D27 and 21.7 mm and 15.9 mm for the isolate D44. Necroses extended into the phloem and xylem as well. There were no symptoms on control trees (Figure 3).

**Figure 4:**
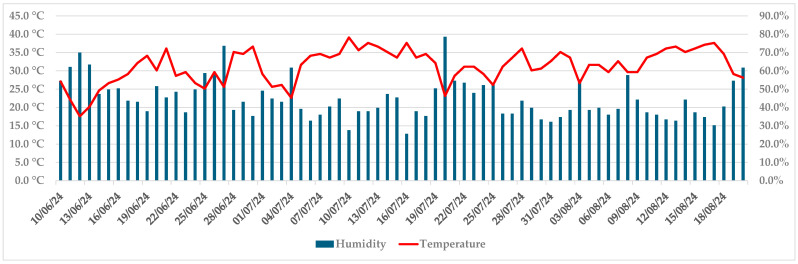
Maximum daily air temperature and humidity during the trial, recorded by an iButton data logger.

Progression of necroses was noticeable up to 1 July, when they achieved on average 19.1 mm in length and 13.2 mm in width (both isolates). In the first week of July, the development of symptoms slowed down, and at this point, the callus formed on some trees. Air temperatures above 31 °C and air humidity below 50% were recorded by an iButton data logger in that period (5–19 July), as well as in two more periods by the end of the trial (26 July–2 August, 10–18 August) (Figure 4).

*D. fraxini* was successfully re-isolated from all inoculated trees. The identity of the obtained isolates was confirmed based on morphological characteristics and molecular analyses of the ITS region of DNA (NCBI accession numbers: PV492180-PV492203). No fungi or oomycetes were obtained from any control tree that remained asymptomatic, and the wound healed.

## 4. Discussion

In this study, a total of 20 *F. angustifolia* trees in eight Croatian sites were analyzed for the presence of *D. fraxini* and other known fungal pathogens to obtain new insight into the etiology of narrow-leaved ash dieback in Croatia more than a decade since the phenomenon was first observed in the country. Symptoms of ash dieback were present and visible in the canopy of the trees in all monitored stands, regardless of the age and other differentiating stand features. Interestingly, although wood necroses or rot were present on the stem base cross-sections of most of the trees, there were no visible outer symptoms on the bark, indicating their possible spread from the root collars and roots. A similar assumption was already reported in an earlier study conducted on *F. angustifolia* [4].

The main finding of this research was the prevalence of *D. fraxini* in the declining trees (found in all locations on 17 trees), which is in accordance with observations reported in several European countries on different ash species [23,24,25,26,28]. For comparison, *H. fraxineus* was detected in the crowns of only two trees in one site. *Diplodia fraxini* was mostly associated with shoots and branches exhibiting sunken cankers and wedge-shaped necroses visible on the cross-sections, which is consistent with reports from other European countries [24,25,28]. However, this research revealed that *D. fraxini* can also be associated with callused canker formations and circle-shaped wood necroses visible on the cross-sections of affected shoots and branches and on wood necroses in stem bases of declining trees. The latter is, to the best knowledge of the authors, the first such finding on *F. angustifolia*. A similar discovery was reported only on *F. excelsior* in Germany, where the fungus was frequently isolated from stem collar necroses easily observable on trees from the outside, in the form of discolored, sunken, or ruptured bark at the stem bases [14,15,29,30]. In this study, *D. fraxini* was confirmed in the stem bases of only two sampled trees in different and mutually distant forest stands, at 60 and 62 years old. The trees in question exhibited significantly defoliated crowns (50% and 70%), with sunken cankers positive to *D. fraxini*, and noticeable wood necroses on stem base cross-sections (90% and 30% of surface); however, they were without any outer symptoms visible on the bark at their stem bases. These differences in results compared with German studies could be due to the methodology employed (sampling at different distances from the ground level), different habitat conditions and forest stand features, or different host species. Previous research has already revealed differences in associated fungal communities between *F. excelsior* and *F. angustifolia* and the impact of various environmental and stand factors (tree age, share of ash in a forest stand mixture, etc.) on the ash dieback progress and fungal species involved [4,13,26,43,44]. The lower parts of the stem, root collars, and roots of *F. angustifolia* should be analyzed in future studies to determine whether the incidence of *D. fraxini* is higher in these plant tissues and comparable with the reported occurrence of the fungus in stem collars of *F. excelsior* in Germany. Nevertheless, the pathogenicity of *D. fraxini* towards *F. angustifolia* was clearly confirmed in this study. Symptoms developed in a relatively short period of time (less than two weeks) on stems of healthy and well-developed six-year-old trees, which occurred under environmental conditions (high temperatures and low humidity) not optimal for *D. fraxini*, which is a mesophilic fungus that does not grow at 35 °C [21]. Although the progression of symptoms slowed down due to climatic conditions and some of the inoculated trees formed a callus around the developed necrosis, the fungus was successfully re-isolated from all the trees, indicating its high survivability in infected plant tissue. Necroses were greater in size than those reported on younger *F. excelsior* plants in similar trials [24,25], suggesting that *D. fraxini* is potentially more aggressive to *F. angustifolia*. This hypothesis should be further tested. In the only similar trial conducted on *F. angustifolia* (in Spain), detached branches were used instead of live plants [23], which is most probably the reason why significantly larger necroses developed in comparison with this study.

Other two species, *D. seriata* and *B. dothidea* from the family *Botryosphaeriaceae*, were isolated from declining *F. angustifolia* trees, although with a low frequency. This differs from what was previously observed for *F. excelsior* and *F. ornus* in Italy and Slovenia [21,24,25,26,27,28]. These differences may depend on various factors, including habitat conditions, forest stand features, host susceptibility, and geographical distribution of the pathogens. In particular, the geographical distribution of *Diplodia subglobosa* appears very limited, although in an expansion phase [21,24].

The second most abundant fungal species isolated was *Diaporthe eres*, detected in the necrotic shoots and branches (crowns), in 14 of 20 sampled trees in seven locations studied. Although the fungus has already been reported as a frequent inducer and colonizer of bark and wood necroses on shoots and branches of *F. excelsior* in several European countries, it demonstrated weak pathogenicity towards ash in artificial trials [18,19,20,24,25]. Given the latter, and the fact that it has been confirmed as an endophyte in *F. excelsior* leaves and twigs [45], it could be considered as an opportunistic pathogen of ash. This remains to be proven for *F. angustifolia* in future studies.

*Hymenoscyphus fraxineus* was found on only seven trees in four locations and in a rather lower number of samples compared with *D. fraxini* and *D. eres.* Although this could be partially attributed to its slow growth in culture, studies in Germany, Italy, and Slovenia on *F. excelsior* have also revealed similar results [15,24,25]. Its association with symptoms in the crown was confirmed in only two 20-year-old *F. angustifolia* trees in location L2, indicating that younger trees are more susceptible. However, this must be further tested in future studies on a greater number of younger *F. angustifolia* trees. *Hymenoscyphus fraxineus* was more frequently isolated from stem bases than shoots and branches (six trees, four locations), often together with *D. fraxini*, *A. gallica*, or *L. tigrinus*, confirming the findings from a previous study on *F. angustifolia* [4] and similar research on *F. excelsior* [15,30]. This result could derive from the fact that *H. fraxineus* is a leaf litter fungus and is therefore able to reach the stem base more easily, unlike *D. fraxini*, which produces pycnidia and ascomata in the cankers on branches and stems [15,24,25].

Two white-rot wood-decaying fungal species, *A. gallica* and *L. tigrinus*, were confirmed in the stem bases of declining *F. angustifolia* trees (three and two trees, respectively). As already mentioned, they were present in trees without any outer bark symptoms of inner wood necroses or decay, indicating their spread from the roots or root collars. The exception was tree No.2 in location L4, the only tree in the study with an open wound at the stem base, where only *A. gallica* was present in the stem base. *A. gallica* was already reported on declining *F. excelsior*, where it is considered to act as a secondary pathogen [16,17,46].

## 5. Conclusions

Overall, *F. angustifolia* dieback in Croatia can be attributed to the synergistic activity of different pathogens, with *D. fraxini* currently acting as the main causative agent. This finding corroborates recent studies conducted in neighboring countries [24,25]. *Diplodia fraxini* was the most frequently isolated fungus from symptomatic shoots and branches on trees of different age, exhibiting different levels of ash dieback symptoms, in all the sites studied. Its ability to cause severe symptoms on *F. angustifolia* was confirmed in a pathogenicity trial.

To the best of our knowledge, this is the first official report of *D. fraxini* in wood necrosis in basal parts of *F. angustifolia*. However, the fungus was not frequent in these tissues, and more detailed studies are necessary in the future to clear its role in the etiology of symptoms in roots and stem bases of declining trees.

Other *Botryosphaeriaceae* species were not frequently isolated, and they seem not to play a major role in the narrow-leaved ash dieback at the moment. It is important to note that *Diplodia subglobosa*, an abundant species on *F. excelsior* in Italy and Slovenia [24,25], was not isolated in this study.

Preliminary results indicate that high temperatures and low air humidity affect the development of *D. fraxini*-induced symptoms in trees, although with no impact on the survival of the fungus in colonized tissues. There is ongoing research to test this further and compare the survival of *D. fraxini* in infected tissues with respect to those of *H. fraxineus*.

## Figures and Tables

**Table 1:** Sampling sites and number of samples collected and analyzed.

Sampling Site	Share of *F. angustifolia* in Total Wood Stock (%)	Age (Years)	Height Above Sea Level (m)	Sampling Date	No. of Sampled Trees	No. of Sampled Shoots and Branches Per Tree	No. of Subsamples Taken from Each Stem Base Cross-Section
L1 Strizivojna 10a	80	62	83	11 October 2021	4	7	5
						7	4
						10	2
						10	4
L2 Strizivojna 21c	95	20	83	28 October 2021	3	8	1
						7	1
						9	3
L3 Kutina 19a	97	25	95	11 July 2023	3	9	3
						9	5
						7	2
L4 Radinje 16c	45	26	88	26 October 2023	2	8	4
						7	5
L5 Radinje 49c	99	70	0	27 November 2023	2	8	1
						7	1
L6 Sunja 53d	79	35	94	6 November 2024	2	10	5
						7	5
L7 Sunja 39b	92	68	94	8 December 2024	2	7	1
						8	2
L8 Mirna 4d	96	60	14	27 March 2023	2	10	5
						7	4

**Table 2:** Accession numbers deposited in GenBank and number of isolates of each fungal species obtained from shoots/branches and from stem bases of the monitored trees.

Fungal Species	Accession No.	Positive Samples		No. of Trees	No. of Sites
		Shoots/Branches	Stem Base		
*Diplodia fraxini*	PV490087	65	5	17	8
*Diaporthe eres*	PV492149	33	-	14	7
*Hymenoscyphus fraxineus*	PV492150	4	6	7	4
*Armillaria gallica*	PV492157	-	9	3	3
*Lentinus tigrinus*	PV492168	-	7	2	1
*Diplodia seriata*	PV492152	1	1	2	2
*Botryosphaeria dothidea*	PV492159	1	-	1	1

**Table 3:** Average lengths (in red) and widths (in blue) of necrosis detected on *F. angustifolia* trees inoculated with *D. fraxini*. Data of necrotic lesions are expressed in mm. The numbers in brackets are the number of days after inoculation.

Monitoring Dates:	24.6 (20)	1.7 (28)	8.7 (35)	15.7 (42)	22.7 (49)	29.7 (56)	5.8 (63)	12.8 (70)	19.8 (77)
Isolate D27	15.6/10.7	18.7/13.3	19.9/14.0	20.3/14.4	20.6/15.0	20.9/15.3	21.4/15.7	21.8/16.0	22.4/16.4
Isolate D44	15.4/11.3	19.4/13.1	19.7/14.0	20.3/15.0	20.7/15.1	21.0/15.4	21.3/15.9	21.5/15.9	21.7/15.9

## Data Availability

Data are contained within the article.

## Abbreviations

The following abbreviations are used in this manuscript:

MEA	Malt extract agar
DNA	Deoxyribonucleic acid
MEB	Malt extract broth
PDA	Potato dextrose agar
ICP-Forests	International Co-operative Programme on Assessment and Monitoring of Air Pollution Effects on Forests
ITS	Internal transcribed spacer
PCR	Polymerase chain reaction
dNTPs	Deoxynucleotide triphosphates
NCBI	National Center for Biotechnology Information
BLAST	Basic Local Alignment Search Tool
DBH	Diameter at breast height

## Appendix A

**Table A1:** Observed symptoms on sampled trees and occurrence of *D. fraxini* and other identified fungi in collected samples (HF = *Hymenoscyphus fraxineus*, DE = *Diaporthe eres*, AG = *Armillaria gallica*, LT = *Lentinus tigrinus*, DS = *Diplodia seriata*, BD = *Botryosphaeria dothidea*).

Location (Forest Stand)	Tree	Height (m)/DBH (cm)	Crown Defoliation (%)	Number of Shoots and Branches Positive to *D. fraxini*	Observed Symptoms on a Stem Base Cross-Section	Number of Cross-Section Wood Sub-Samples Positive to *D. fraxini*	Other Fungi Present in Sampled Trees
L1 Strizivojna 10a	1	28.4/35	45	5/7	rot and necrosis on 40% of surface	0/5	DE in crown HF in stem base
	2	29.1/34	55	0/7	four smaller individual necroses	0/4	DS in stem base
	3	28.2/35	60	8/10	central irregular necrosis on 20% of surface	0/2	DE in crown
	4	27.9/36	70	6/10	rot and necrosis on 30% of surface	1/4	DE in crown HF in stem base
L2 Strizivojna 21c	1	15.3/14	30	2/8	none	0/1	HF in crown
	2	15.1/15	20	1/7	none	0/1	-
	3	15.6/15	40	0/9	three individual necroses on 15% of surface	0/3	HF in both crown and stem base AG in stem base
L3 Kutina 19a	1	19.7/21	30	3/9	rot and necrosis on 20% of surface	0/3	DE in crown
	2	14.3/16	70	2/9	multiple necroses on 25% of surface	0/5	DE and BD in crown
	3	18.4/19	15	0/7	small necroses on 5% of surface	0/2	DE in crown
L4 Radinje 16c	1	14.8/15	25	2/8	central irregular discoloration on 50% of surface	0/4	DS in crown
	2	13.9/14	45	2/7	rot and necrosis on 80% of surface	0/5	DE in crown AG in stem base
L5 Radinje 49c	1	24.7/33	50	3/8	small central discoloration on 5% of surface	0/1	DE in crown
	2	24.9/34	30	2/7	small central discoloration on 3% of surface	0/1	-
L6 Sunja 53d	1	26.2/36	20	7/10	rot and necrosis on 70% of surface	0/5	DE in crown HF and LT in stem base
	2	23.5/35	55	4/7	rot and necrosis on 30% of surface	0/5	DE in crown HF and LT in stem base
L7 Sunja 39b	1	28.3/34	65	4/7	one small central necroses on 3% of surface	0/1	DE in crown
	2	28.4/42	35	3/8	two small necroses on 5% of surface	0/2	DE in crown
L8 Mirna 4d	1	29.8/28	50	8/10	rot and necrosis on 90% of surface	2/5	DE in crown HF and AG in stem base
	2	27.4/27	40	3/7	four small necroses on 5% of surface	0/4	DE in crown

## References

[1] Boshier D., Cordero J., Harris S., Pannell J., Rendell S., Savill P., Stewart J., Cundall N., Hubert J., Samuel S. (2005). Ash Species in Europe: Biological Characteristics and Practical Guidelines for Sustainable Use.

[2] Hill L., Jones G., Atkinson N., Hector A., Hemery G., Brown N. (2019). The £ 15 Billion Cost of Ash Dieback in Britain. Curr. Biol..

[3] Kowalski T. (2006). *Chalara fraxinea* sp. nov. Associated with Dieback of Ash (*Fraxinus excelsior*) in Poland. For. Pathol..

[4] Kranjec Orlović J., Moro M., Diminić D. (2020). Role of Root and Stem Base Fungi in *Fraxinus angustifolia* (Vahl) Dieback in Croatian Floodplain Forests. Forests.

[5] Carroll D., Boa E. (2024). Ash Dieback: From Asia to Europe. Plant Pathol..

[6] Kabiljo M., Bobinac M., Andrašev S., Milenković I., Šušić N. (2025). The Importance of Stand Structure in Narrow-Leaved Ash (*Fraxinus angustifolia* Vahl) Dieback—Insights from an Extensively Managed Stand on a Humogley Soil in Serbia. Forests.

[7] Bengtsson S.B.K., Barklund P., von Brömssen C., Stenlid J. (2014). Seasonal Pattern of Lesion Development in Diseased *Fraxinus excelsior* Infected by *Hymenoscyphus pseudoalbidus*. PLoS ONE.

[8] Gross A., Holdenrieder O., Pautasso M., Queloz V., Sieber T.N. (2014). *Hymenoscyphus pseudoalbidus*, the Causal Agent of E Uropean Ash Dieback. Mol. Plant Pathol..

[9] Zhao Y.-J., Hosoya T., Baral H.-O., Hosaka K., Kakishima M. (2013). *Hymenoscyphus pseudoalbidus*, the Correct Name for Lambertella Albida Reported from Japan. Mycotaxon.

[10] Nielsen L.R., McKinney L.V., Hietala A.M., Kjær E.D. (2017). The Susceptibility of Asian, European and North American *Fraxinus* Species to the Ash Dieback Pathogen *Hymenoscyphus fraxineus* Reflects Their Phylogenetic History. Eur. J. For. Res..

[11] Coker T.L., Rozsypálek J., Edwards A., Harwood T.P., Butfoy L., Buggs R.J. (2019). Estimating Mortality Rates of European Ash (*Fraxinus excelsior*) under the Ash Dieback (*Hymenoscyphus fraxineus*) Epidemic. Plants People Planet.

[12] Bakys R., Vasiliauskas A., Ihrmark K., Stenlid J., Menkis A., Vasaitis R. (2011). Root Rot, Associated Fungi and Their Impact on Health Condition of Declining *Fraxinus excelsior* Stands in Lithuania. Scand. J. For. Res..

[13] Lenz H.D., Bartha B., Straßer L., Lemme H. (2016). Development of Ash Dieback in South-Eastern Germany and the Increasing Occurrence of Secondary Pathogens. Forests.

[14] Meyn R., Langer G.J., Gross A., Langer E.J. (2019). Fungal Colonization Patterns in Necrotic Rootstocks and Stem Bases of Dieback-Affected *Fraxinus excelsior* L. For. Pathol..

[15] Peters S., Fuchs S., Bien S., Bußkamp J., Langer G.J., Langer E.J. (2023). Fungi Associated with Stem Collar Necroses of *Fraxinus excelsior* Affected by Ash Dieback. Mycol. Prog..

[16] Enderle R., Peters F., Nakou A., Metzler B. (2013). Temporal Development of Ash Dieback Symptoms and Spatial Distribution of Collar Rots in a Provenance Trial of *Fraxinus excelsior*. Eur. J. For. Res..

[17] Spiegel P., Hintze T., Kopp A., Sahli M., Detter A., Queloz V., Prospero S., Heinzelmann R. (2025). Synergistic Negative Effects of Ash Dieback and Armillaria Root Rot on Health and Stability of Mature Ash Trees. For. Ecol. Manag..

[18] Kowalski T., Kraj W., Bednarz B. (2016). Fungi on Stems and Twigs in Initial and Advanced Stages of Dieback of European Ash (*Fraxinus excelsior*) in Poland. Eur. J. For. Res..

[19] Kowalski T., Bilański P., Kraj W. (2017). Pathogenicity of Fungi Associated with Ash Dieback towards *Fraxinus excelsior*. Plant Pathol..

[20] Vemić A., Tomšovský M., Jung T., Milenković I. (2019). Pathogenicity of Fungi Associated with Ash Dieback Symptoms of One-Year-Old *Fraxinus excelsior* in Montenegro. For. Pathol..

[21] Alves A., Linaldeddu B.T., Deidda A., Scanu B., Phillips A. (2014). The Complex of *Diplodia* Species Associated with *Fraxinus* and Some Other Woody Hosts in Italy and Portugal. Fungal Divers..

[22] Saccardo P.A. (1884). Sylloge Fungorum Omnium Hucusque Cognitorum.

[23] Elena G., León M., Abad-Campos P., Armengol J., Mateu-Andrés I., Güemes-Heras J. (2018). First Report of *Diplodia fraxini* Causing Dieback of *Fraxinus angustifolia* in Spain. Plant Dis..

[24] Linaldeddu B.T., Bottecchia F., Bregant C., Maddau L., Montecchio L. (2020). *Diplodia fraxini* and *Diplodia subglobosa*: The Main Species Associated with Cankers and Dieback of *Fraxinus excelsior* in North-Eastern Italy. Forests.

[25] Linaldeddu B.T., Bregant C., Montecchio L., Brglez A., Piškur B., Ogris N. (2022). First Report of *Diplodia fraxini* and *Diplodia subglobosa* Causing Canker and Dieback of *Fraxinus excelsior* in Slovenia. Plant Dis..

[26] Benigno A., Bregant C., Aglietti C., Rossetto G., Tolio B., Moricca S., Linaldeddu B.T. (2023). Pathogenic Fungi and Oomycetes Causing Dieback on *Fraxinus* Species in the Mediterranean Climate Change Hotspot Region. Front. For. Glob. Change.

[27] Cimmino A., Maddau L., Masi M., Linaldeddu B.T., Pescitelli G., Evidente A. (2017). Fraxitoxin, a New Isochromanone Isolated from *Diplodia fraxini*. Chem. Biodivers..

[28] Benigno A., Aglietti C., Rossetto G., Bregant C., Linaldeddu B.T., Moricca S. (2023). *Botryosphaeriaceae* Species Associated with Stem Canker, Shoot Blight and Dieback of *Fraxinus ornus* in Italy. Forests.

[29] Langer G. (2017). Collar Rots in Forests of Northwest Germany Affected by Ash Dieback. Balt. For..

[30] Peters S., Gruschwitz N., Bien S., Fuchs S., Bubner B., Blunk V., Langer G.J., Langer E.J. (2024). The Fungal Predominance in Stem Collar Necroses of *Fraxinus excelsior*: A Study on *Hymenoscyphus fraxineus* Multilocus Genotypes. J. Plant Dis. Prot..

[31] Čavlović J. (2010). Prva Nacionalna Inventura Šuma Republike Hrvatske.

[32] Potočić N., Seletković I., Jakovljević T., Marjanović H., Indir K., Medak J., Ognjenović M., Zorić N. (2020). Oštećenost Šumskih Ekosustava Republike Hrvatske—Izvješće Za 2019. Godinu.

[33] Potočić N., Seletković I., Medak J., Jakovljević T., Marjanović H., Indir K., Marušić M., Zorić N., Bogdanić R., Lovrić V. (2025). Oštećenost Šumskih Ekosustava Republike Hrvatske—Izvješće Za 2024. Godinu.

[34] Barić L., Županić M., Pernek M., Diminić D. (2012). First Records of *Chalara fraxinea* in Croatia–a New Agent of Ash Dieback (*Fraxinus* Spp.). Šumar. List.

[35] Anić I. (2001). Uspijevanje i Pomlađivanje Sastojina Poljskog Jasena (*Fraxinus angustifolia* Vahl) u Posavini. Ph.D. Thesis.

[36] Eichhorn J., Roskams P., Potocic N., Timmermann V., Ferretti M., Mues V., Szepesi A., Durrant D., Seletkovic I., Schroeck H.-W. (2016). Part IV Visual Assessment of Crown Condition and Damaging Agents. Manual on Methods and Criteria for Harmonized Sampling, Assessment, Monitoring and Analysis of the Effects of Air Pollution on Forests.

[37] Cenis J.L. (1992). Rapid Extraction of Fungal DNA for PCR Amplification. Nucleic Acids Res..

[38] Kranjec Orlović J., Diminić D., Ištok I., Volenec I., Hodak L., Grubešić M., Tomljanović K. (2024). Fungal Presence and Changes of Wood Structure in Bark Stripping Wounds Made by Red Deer (*Cervus elaphus* L.) on Stems of *Fraxinus angustifolia* (Vahl). Forests.

[39] Gardes M., Bruns T.D. (1993). ITS Primers with Enhanced Specificity for Basidiomycetes—Application to the Identification of Mycorrhizae and Rusts. Mol. Ecol..

[40] White T.J., Bruns T., Lee S., Taylor J. (1990). Amplification and Direct Sequencing of Fungal Ribosomal RNA Genes for Phylogenetics. PCR Protocols: A Guide to Methods and Applications.

[41] Hall T.A. (1999). BioEdit: A User-Friendly Biological Sequence Alignment Editor and Analysis Program for Windows 95/98/NT. Nucleic. Acids Symp. Ser..

[42] Altschul S.F., Gish W., Miller W., Myers E.W., Lipman D.J. (1990). Basic Local Alignment Search Tool. J. Mol. Biol..

[43] Grosdidier M., Scordia T., Ioos R., Marçais B. (2020). Landscape Epidemiology of Ash Dieback. J. Ecol..

[44] Madsen C.L., Kosawang C., Thomsen I.M., Hansen L.N., Nielsen L.R., Kjær E.D. (2021). Combined Progress in Symptoms Caused by *Hymenoscyphus fraxineus* and *Armillaria* Species, and Corresponding Mortality in Young and Old Ash Trees. For. Ecol. Manag..

[45] Barta M., Pastirčáková K., Ostrovský R., Kobza M., Kádasi Horáková M. (2022). Culturable Endophytic Fungi in *Fraxinus excelsior* and Their Interactions with *Hymenoscyphus fraxineus*. Forests.

[46] Skovsgaard J., Thomsen I., Skovgaard I., Martinussen T. (2010). Associations among Symptoms of Dieback in Even-aged Stands of Ash (*Fraxinus excelsior* L.). For. Pathol..

